# A Microcontroller-Based Device for Liquid Intake and Lick Analysis in Rodents

**DOI:** 10.3390/bios16050268

**Published:** 2026-05-06

**Authors:** Sikandar Ali, Massimo Ubaldi, Sofia Christina Gkolfinopoulou, Andrea Della Valle, Fabio Casarola, Adolfo Russo, Matteo Piersantelli, Roberto Ciccocioppo

**Affiliations:** 1 School of Pharmacy, Center for Neuroscience, Pharmacology Unit, University of Camerino, 62032 Camerino, Italy; sofia.gkolfinopoulou@unicam.it (S.C.G.); andrea.dellavalle@unicam.it (A.D.V.); 2 School of Advanced Studies, University of Camerino, 62032 Camerino, Italy; massimo.ubaldi@unicam.it; 3 AM Microsystems Srl, 62100 Macerata, Italy; f.casarola@am-microsystems.com (F.C.); a.russo@am-microsystems.com (A.R.); m.piersantelli@am-microsystems.com (M.P.)

**Keywords:** microcontroller, lickometer, liquid intake monitoring, behavioral neuroscience, rodent behavior, ingestive behavior, IOT, embedded system, sensor technology

## Abstract

Drinking behavior is an important parameter in laboratory animal experiments. Traditionally, the amount of fluid consumed is recorded manually by the experimenter, who weighs the bottle at predetermined timepoints to calculate the intake. This approach is labor-intensive and involves human intervention, which can disturb the animals and affect their natural drinking patterns. Automated drinkometer systems have been developed to enable continuous and automated recording of fluid consumption. One advantage of these devices is that they allow researchers not only to measure total fluid intake but also to analyze the microstructure of drinking behavior (e.g., number of licks, bouts, and inter-bout intervals). However, these systems typically require dedicated cages equipped with proprietary drinkometers, making them highly expensive and often unsuitable for monitoring fluid consumption under standard home cage conditions. Here, we describe the development of a new cost-effective microcontroller-based device for continuous monitoring of liquid consumption and analysis of licking microstructure, which can be integrated with existing cages. The system is based on capacitive sensing technology for the assessment of liquid consumption. A customized bottle handler and capacitive sensor have been designed, which can be mounted on a standard 250 mL sized bottle for data acquisition. The device has been tested both in mice and in rats. The results demonstrated that for both animal species the system reproducibly and reliably recorded fluid consumption, also allowing for licking analysis. The data are acquired autonomously through custom devices and directly transferred to a computer for storage and analysis.

## 1. Introduction

The assessment of liquid consumption is very important in research and in laboratory animal facilities to control animal health and to monitor many experimental data including physiological, metabolic, neurologic, endocrine, and behavioral parameters [1,2,3]. In most laboratories, fluid consumption is routinely measured by weighing the fluid container (i.e., bottle) or by monitoring the consumption through graduate tubes or burettes at the beginning of the experiments and at any other established timepoint. Fluid intake is determined by calculating the difference between the initial and final recorded volume. These manual approaches are labor-intensive and do not enable continuous or high-resolution monitoring of fluid intake. Moreover, they need the intervention of a human operator that could disturb normal animal activity, which would result in triggering stress and anxiety that could be detrimental in many behavioral experiments [4].

The assessment of fluid intake is particularly important in preclinical research. A prototypical example comes from the alcohol research field, where under a free-choice drinking paradigm, the amount of alcohol solution consumed by an animal over time is measured at specific intervals [5]. Typically, this assessment is performed manually by weighing the bottle containing the alcohol solution. However, during this process, handling or moving the bottle may disturb the animals and alter their drinking behavior, potentially introducing measurement errors, especially in small rodents such as mice, which consume relatively small volumes of fluid. In studies where pharmacological agents are administered to manipulate (e.g., reduce) alcohol intake, it becomes impossible to determine whether the treatment affects not only the total amount consumed but also the microstructure of drinking behavior, such as the latency to the first drinking bout, the number and duration of bouts, or the number of licks. These parameters can provide valuable insights into the mechanisms of action of the tested drug. In addition, manual data recording, being discontinuous, does not allow for retrospective analysis using timepoints different from those established at the beginning of the study. To obtain this additional information, automated drinkometer systems have been developed. Several such devices are commercially available; however, they are generally expensive, require dedicated monitoring cages, and cannot be easily adapted to the standard Plexiglas home cages commonly used for housing laboratory rodents. Continuous and automated home cage monitoring of fluid (e.g., water) intake would also be highly valuable for managing large laboratory animal breeding facilities. Using fluid intake as a proxy, this approach enables constant assessment of the animals’ health directly within their home cages, something that is difficult to achieve through manual methods [6,7]. Under standard housing conditions, baseline water consumption in laboratory mice typically ranges from 3 to 7 mL per day [8], whereas adult rats generally consume approximately 9–12 mL of water per 100 g of body weight per day, corresponding to about 21–28 mL/day for rats weighing 228–240 g [9].

Fluid consumption levels, as well as drinking patterns, can be significantly altered in the presence of pathological conditions or as a consequence of pharmacological manipulations [10]. Moreover, monitoring water intake enables the detection of pregnancies and parturition, as these physiological states are typically associated with marked and rapid changes in the dams’ drinking behavior [11].

**Figure 1:**
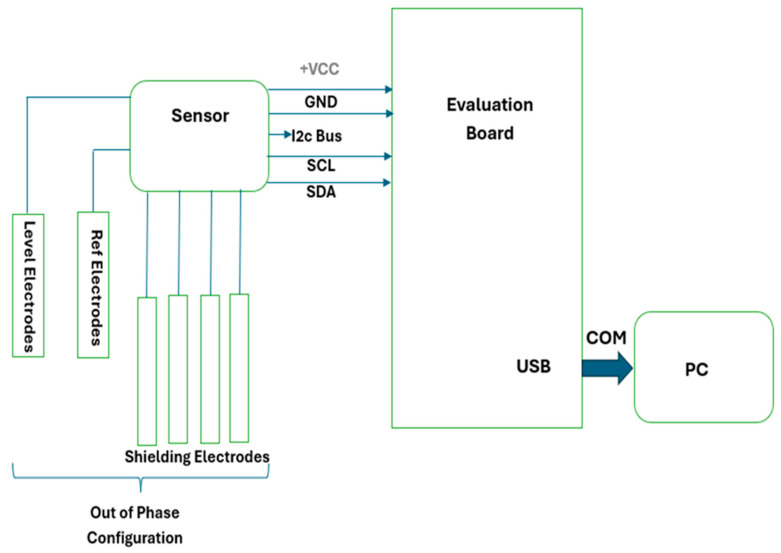
Methodology of proposed work.

Here we describe the development of a new cost-effective device for continuous monitoring of liquid consumption and analysis of licking microstructure, which can be integrated with the existing Plexiglas cage currently used for laboratory rodents. This system is based on a capacitive sensing detector which can be mounted on a standard 250 mL sized bottle for data acquisition. The data are acquired autonomously and directly transferred to a storage device, for example, a PC (Figure 1).

## 2. Materials and Methods

The proposed system uses an FDC1004 capacitive sensor (Texas Instrument, USA) which has 4-channel capacitances to a digital converter for implementing capacitive sensing. The system consumes low power, has a high resolution, and gives a low-cost solution to measure the liquid level without contacting the liquid present in the container. Experimental tests were performed in a ventilated room of the animal facility at the School of Pharmacy, University of Camerino. The room was held at a controlled temperature of 22 °C and humidity (30–40% RH relative humidity). The Standard GM500 and the 1500U Tecniplast cages (Tecniplast, IT) were used for mice and for rats, respectively. Cages were equipped with a standard food pellet container and dust free wooden litter. The bottles used for experiments were the standard 250 mL capacity, square in shape, and with a stainless-steel spout. To validate the efficiency of the device, 2 adult (>8 weeks) male CD1 mice, weighing 34 g and 36 g, and 2 adult Wistar rats (>8 weeks), weighing 228–235 g (Charles River Laboratories, IT), were commonly housed and tested. Animals were kept on a 12 h dark/light cycle from (9:00–21:00), were maintained with standard food pellets (Standard Diet-Mucedola Settimo Milanese (MI), Italy) and water was available at libitum through drinking bottles. Tests were performed to monitor the liquid consumption in mice and in rats (5 days for mice and 3 days for rats).

The system (Figure 1) employs capacitive sensors composed of liquid-level electrodes, reference electrodes, and shielding electrodes to measure input signals. The reference electrodes establish a constant reference point for the capacitive sensor. By comparing the capacitance between the reference and sensing electrodes, the sensor can detect environmental changes. The level electrodes measure the capacitance value, while the ratio between the reference and level capacitance enables real-time calculation of the liquid level inside the container. To prevent interference from external electric fields, shielding electrodes isolate the sensing area from the surrounding environment. This shielding ensures that the sensor focuses solely on capacitance changes caused by the target material, minimizing the influence of external factors. The sensor is configured in an Out-of-Phase mode to acquire input data. It connects through a 4-pin connector consisting of a power supply pin (+VCC), ground pin, and two I^2^C communication lines (SCL and SDA). These inputs interface with the evaluation board FDC1004EVM. The I^2^C communication protocol is used for data acquisition. The FDC1004 digital sensor measures capacitance and transmits the data to the evaluation board for further processing. The FDC1004EVM evaluation module includes a USB-to-I^2^C converter based on the MSP430F5528 microcontroller. Using Texas Instruments’ dedicated software, register configurations are set through the FDC1004, and data are transmitted to a PC terminal via USB communication. The sensor’s data acquisition rate is 400 samples per second.

### 2.1. Coplanar Configuration

An electric field is produced by coplanar arrangements of electrodes at which potential difference is applied. This electric field extends as a fringing effect some distance in addition to existing immediately between the conductive plates. Any type of material could be applied between two electrodes by using this setup. Any changes in dielectric property have an impact on the inter-electrode capacitance reading because the complainer plates fringing electric fields permeate the material.

The two-dimensional electric filed distribution in this electrode’s setup can be solved by applying the inverse discrete cosine transform, which yields the coordinate Z = (x + iy) in the rectangular x-y plane and converted to W = u(x,y) + iv(x,y) in the inverse cosine u-v plane.

So, from the inverse cosine transform:

(1) $$W=V_{0}-\frac{V_{0}}{\pi }\cos^{-1}\left(\frac{2z}{a}\right)$$

We can write:

(2) $$x=\frac{a}{2}\cos\left[\frac{\pi }{V_{0}}\left(V_{0}-W\right)\right]\;\to \;x+iy=\frac{a}{2}\cos\left[\frac{\pi }{V_{0}}\left(V_{0}-U\right)-i\frac{\pi }{V_{0}}v\right]$$

So basically, this equation allows us to obtain the value of x and y coordinates in terms of u and v:

(3) $$x=\frac{a}{2}\cos\left[\frac{\pi }{v_{n}}\left(V_{0}-u\right)\right]\cosh\left(\frac{\pi v}{v_{0}}\right)$$

(4) $$y=\frac{a}{2}\sin\left[\frac{\pi }{v_{0}}\left(V_{0}-u\right)\right]\sinh\left(\frac{\pi v}{v_{0}}\right)$$

**Figure 2:**
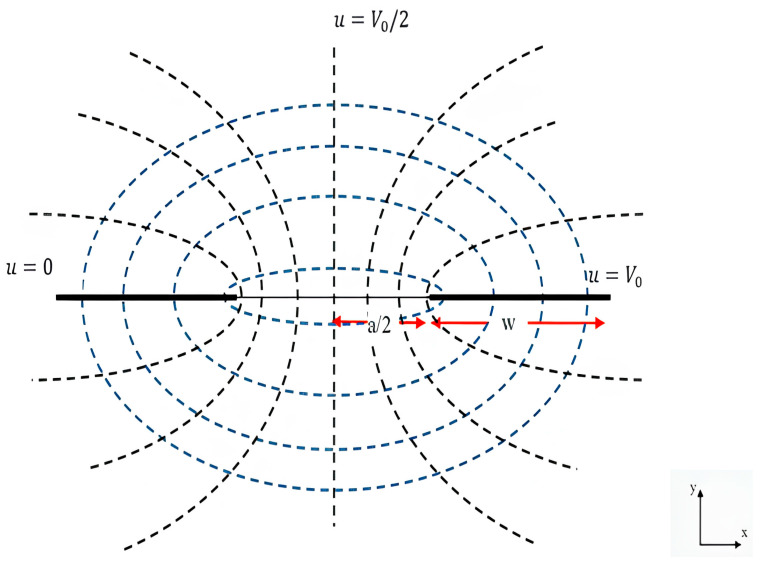
Electric field configuration of two-dimensional coplanar electrodes. Black lines represent equipotential and blue lines represent electric flux lines.

Thus, this gives us the elliptic Equation (5) and hyperbolic Equation (6), which refer to the field line as shown in (Figure 2):

(5) $$\frac{x^{2}}{\cosh^{2}\left(\frac{\pi v}{V_{0}}\right)}+\frac{y^{2}}{\sinh^{2}\left(\frac{\pi v}{V_{0}}\right)}=\frac{a^{2}}{4}$$

(6) $$\frac{x^{2}}{\cos^{2}\left[\frac{\pi }{V_{0}}\left(V_{0}-u\right)\right]}-\frac{y^{2}}{\sin^{2}\left[\frac{\pi }{V_{0}}\left(V_{0}-u\right)\right]}=\frac{a^{2}}{4}$$

It is assumed that every electrical charge is present on the electrode surface (y = 0); since the thickness is negligible in relation to the electrode width, Gauss’s law is used to solve the total electrical charge.

(7) $$Q=\iint D\left(y=0\right)dA$$

Here, in Equation (7), electrical displacement vector is defined as:

(8) $$D\left(y=0\right)=-\varepsilon_{r}\varepsilon_{0}{\left(\frac{\delta u}{\delta y}\right)}_{y=0}$$

The boundary condition of u = V_0_ at the surface w on the plane y = 0 gives:

(9) $${\left(\frac{\delta u}{\delta y}\right)}_{y=0}=-\frac{2V_{0}}{a\pi \;{\sinh\left(\frac{\pi v}{V_{0}}\right)}_{y=0}}$$

So, from Equation (8), this gives us:

(10) $$D\left(y=0\right)=\varepsilon_{r}\varepsilon_{0}\;\frac{2v_{s}}{a\pi \;{\sinh\left(\frac{\pi v}{V_{0}}\right)}_{y=0}}$$

And, solving for QQ, the Gauss’s law we obtain is as follows:

(11) $$Q=\frac{2\varepsilon_{r}\varepsilon_{0}iv_{0}}{\pi }\ln\left[\left(1+\frac{2w}{a}\right)+\sqrt{{\left(1+\frac{2w}{a}\right)}^{2}-1}\right]$$

Hence the capactiance expressed by C = $\frac{Q}{V0}$ is:

(12) $$C=\frac{2\varepsilon_{r}\varepsilon_{\vartheta }i}{\pi }\ln\left[\left(1+\frac{2w}{a}\right)+\sqrt{{\left(1+\frac{2w}{a}\right)}^{2}-1}\right]$$

The maximal field penetration depth, T along the Y direction, is determined by the width of the sensing electrodes w. It can be derived from elliptical contours v(x; y). This corresponds to the maximum *y*-axis displacement of the field lines. This provides us with an additional measure of the coplanar electrode pairs sensitivity, which is computed as follows:

$$T=\frac{a}{2}\;\sqrt{{\left(1+\frac{2w}{a}\right)}^{2}-1}$$

Hence, capactiance can be rewritten in terms of penetration depth T.

$$C=\frac{2\varepsilon_{r}\varepsilon_{0}l}{\pi }\;\ln\left[\frac{2}{a}\left(T+\sqrt{T^{2}+1}\right)\right]$$

Here, finally we could calculate the sensitivity of the capacitor along-axis from the changing difference between the liquid inside the bottle and the electrical permittivity.

(13) $$\Delta C=\frac{2\left(\varepsilon_{r}-1\right)\varepsilon_{o}\Delta l}{\pi }\;ln\;\left[\frac{2}{a}\left(T+\sqrt{T^{2}+1}\right)\right]$$

**Figure 3:**
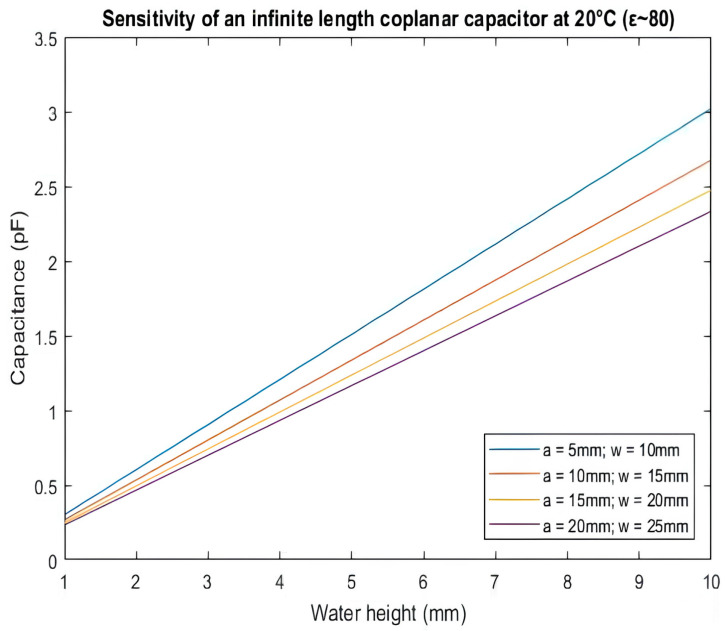
MATLAB (R2021b 9.11) simulation of Equation (13) for capacitance variation as a function of the increase in water height (ε = 80). Different physical values of a and w were taken into account and included in the calculation of penetration depth T.

From the result (Figure 3), it is clear that, to maximize the sensitivity of the device we need to increase the field penetration depth, by increasing the width of electrodes (w) and minimizing the gap separation (a). Considering the dimensions of a standard bottle used in the lab facility, the theory suggests that the best result will be given by physical dimensions of 10 mm for the width of the electrodes and 5 mm for the gap separation between them.

### 2.2. Readout Signal

Usually, capacitive sensing has been implemented through a conventional ratio-metric approach between a ‘level’ electrode pair and a ‘reference’ electrode pair. This is accomplished by using a capacitive-to-digital converter with one of the two electrodes that compose the coplanar capacitor driven to the ground. In this case, the Out of Phase technique has been used (D. Wang—Texas Instruments, Inc., TX, USA) [12], in which the two pairs of electrodes, levels, and references were driven 180° out of phase between two other shielding pairs of electrodes, making it possible to significantly reduce noise from outside sources such as, for example, human hand contact.

The two signals obtained (level and reference) are used to calculate the change in capacitance using the following formula, which is the height of the reference electrode and is the ratio between the two signals when read with the empty container.

(14) $$V=h_{RL}\left[\left(\frac{1}{n}\sum_{i=1}^{n}\frac{Lev_{i}}{Ref_{i}}\right)-\frac{c_{IEVO}}{c_{RLO}}\right]$$

### 2.3. Calibration

To obtain reliable results that ensure agreement between real measurements and the electronic system readout, proper instrument calibration is essential. Depending on the specific application, calibration should emphasize either trueness (accuracy) or precision. A robust strategy involves constructing a calibration curve to characterize the system’s response under controlled conditions.

**Figure 4:**
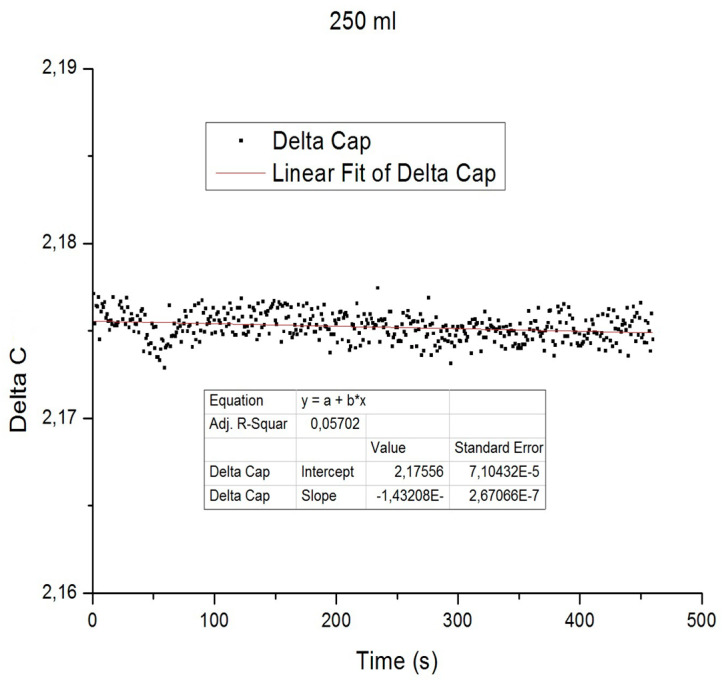
Least squares regression line for a set of data at 250 mL. Image represents a set of standard samples made at steps of 50 mL with this method. An error value was obtained by iterating the experimental measurements at each point.

The capacitive-to-digital converter outputs signals expressed in pF, which are transmitted to a microcontroller via a standard I^2^C protocol. The firmware implements Equation (12) as an algorithm embedded in the microcontroller, returning an adimensional value. These values reflect variations in capacitance and are subsequently converted into volume (mL) through an interpolation function derived from experimental calibration data. Each point of the interpolation function was obtained by recording the y-intercept of a linear regression performed on data acquired over a 5 min interval, allowing stabilization of the readout signal (Figure 4).

This will allow us to reconstruct the interpolation function used as a calibration curve.

(15) $$y=A_{1}e^{-\frac{x}{t_{1}}}+y_{0}$$

**Figure 5:**
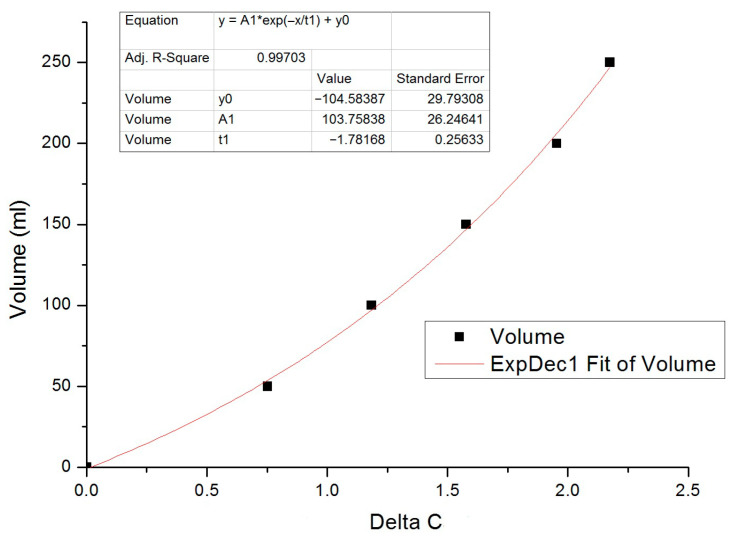
Interpolation function is used as calibration curve to convert signal readout in unit volume.

Using this technique, the system has reached an accuracy of ~1 mL on the signal readout. The final electronic design includes a Bluetooth low-energy module to ensure wireless communication and a wireless recharging system to maintain a compact and sealed device to prevent any liquid leaks from compromising the electronics (Figure 5).

### 2.4. Lickings Microstructure Analysis

Rodents’ fluid-licking behavior is frequently employed in different experimental behavioral scenarios to assess fluid consumption and greed. The repetitive jaw and tongue movements that characterize this behavior are highly stereotyped. This behavior has usually been measured with a lickometer by using either an optical or electrical sensor [13]. Capacitive sensing is an alternative solution that addresses the problem of measuring the liquid intake at the same time. The biggest challenge in doing this is being able to electrically isolate the stainless-steel spout with an insulator. The heat shrink wrap was utilized in this case only for the time needed in this experiment. This type of analysis was tested solely on male CD-1 mice. Achieving a high instrumental sensitivity requires collecting data quickly enough to track a single event. The frequency of licking in C5SBL/6 and DBA/2J mice is 8.5 Hz and 10.6 Hz, respectively [14]. According to J. D. Boughter et al., there are genetic variances in the length of the interlick interval (ILI) that are unrelated to sex, the degree of water deprivation, or the total number of licks. Furthermore, the magnitude of the licking bursts and the intervals between them are significant characteristics or important parameters [15]. To obtain these different parameters, the data acquisition rate should be at least twice the frequency of the analog signal read, as stated by the Nyquist–Shannon sampling theorem, to have a sufficient condition for a sampling rate that allows for a discrete series of samples. Therefore, a sampling period of ≤50 ms has been required.

fsamp ≥ 2 * (fsig)

The system has acquired data samples at the rate of 400 S/s on FDC1004 and multiplied by each acquisition channel. The system has acquired approximately 100 Hz of readout data, which translates to a 10 ms sampling period/time, which is more than adequate for our purposes. Therefore, these parameters have been used to run several 5 min acquisition sessions to trigger the licking behavior and obtain the subsequent outcomes for several sessions.

## 3. Hardware Implementation

**Figure 6:**
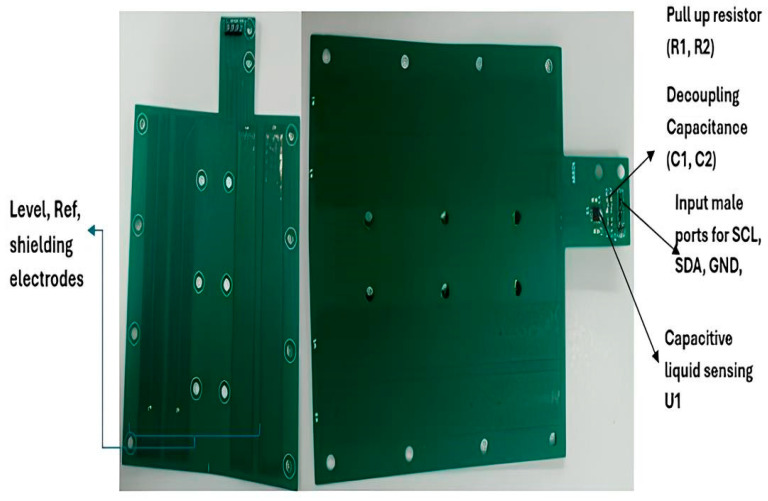
Custom Sensor design for liquid level sensing. The image shows the custom sensor design that has been implemented on a flexible printed circuit board (PCB) to measure the liquid level inside the bottle. The FDC1004 chip has been placed on the board for capacitive liquid sensing and labeled as U1. Two pull-up resistors for I^2^C signals have been mounted on flexible PCB, whereas C3 and C4 are decoupling capacitance. The P2 has 4 male pins for power supply and the I^2^C signals.

**Figure 7:**
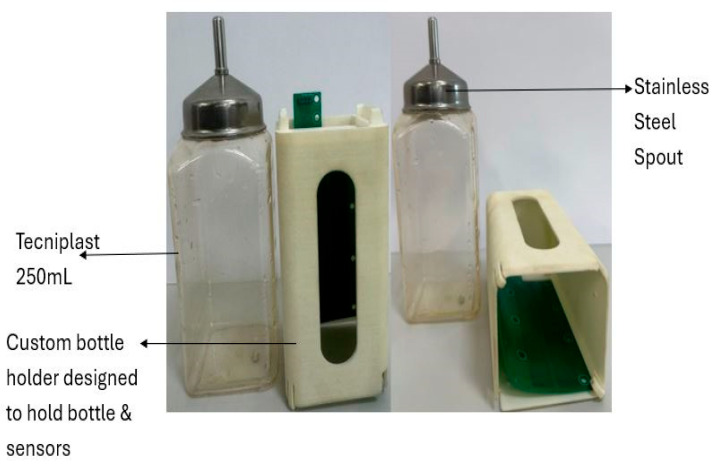
Custom bottle holder for sensor prototype. The figure shows custom-designed bottle holder integrating the liquid container and capacitive sensor. The holder allows secure placement and easy replacement of standard 250 mL laboratory bottles. The sensor, positioned within the holder, measures capacitance variations corresponding to liquid-level changes. A front viewing window enables visual inspection of the bottle, providing secondary confirmation of real-time sensor readings.

A microcontroller-based system was developed to assess liquid consumption in rodents. Extensive research and refinement were carried out to finalize the custom hardware design. The system incorporates an FDC1004 capacitive sensor equipped with level electrodes, reference electrodes, and shielding electrodes. The electrodes are configured in an Out-of-Phase arrangement to enhance measurement accuracy and minimize interference (Figure 6 and Figure 7).

**Figure 8:**
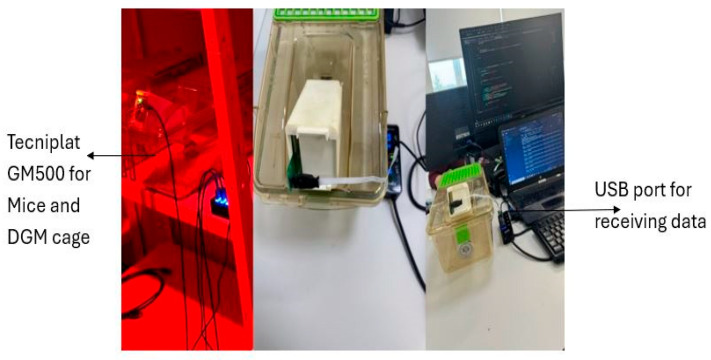
Microcontroller-based liquid consumption system assembly. Figure shows a wired microcontroller-based system to monitor liquid consumption in real time.

In addition, a wired microcontroller-based system was developed to monitor liquid consumption in real time without the need for human intervention or direct contact with the liquid (Figure 8). The system employs a custom capacitive sensor positioned inside a 3D-printed bottle holder, which is mounted on the animal cage. The sensor detects variations in capacitance that correspond to changes in the liquid level within the bottle. The capacitive sensor is interfaced with the FDC1004EVM evaluation board (Texas Instruments, Texas, USA), which communicates with a PC terminal via a Universal Serial Bus (USB) connection using the I^2^C communication protocol. Sensor configuration and register setup were performed using the Texas Instruments graphical user interface (GUI). Once configured, real-time data acquisition was carried out using Termite 3.4 software (CompuPhase, The Netherlands). Termite provides a simple serial communication interface, allowing the user to configure the USB port and baud rate to receive capacitance readings in picofarads (pF). The acquired pF data were converted into liquid volume (mL) using a predetermined calibration function. All recorded data were stored as text files for subsequent analysis. Processed data were visualized and analyzed using OriginPro2021 software (OriginLab Corporation, USA), where capacitance–volume relationships and temporal liquid consumption patterns were plotted and interpreted.

## 4. Experimental Validation

**Figure 9:**
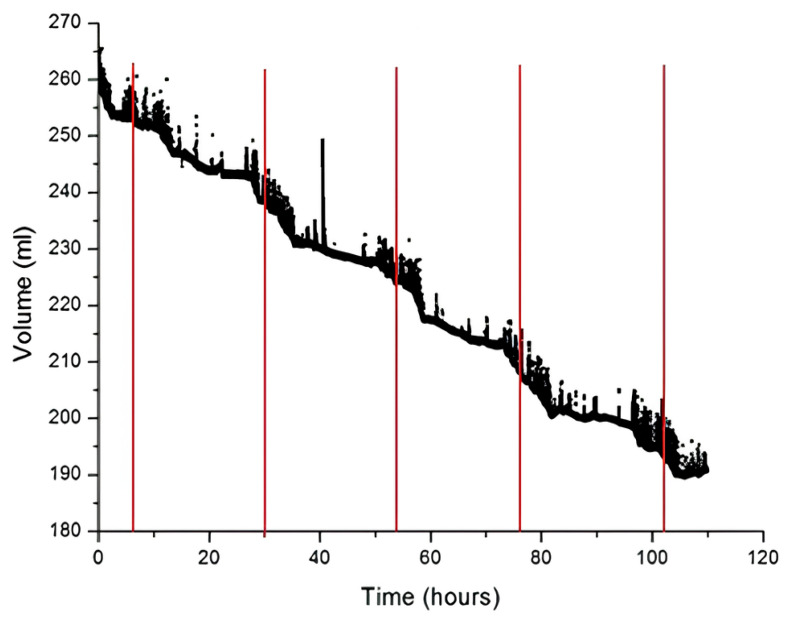
Drinking pattern in mice during the 5 days of testing. Midnight is shown by red vertical lines. The time is plotted on *X*-axis (in hours), whereas on *Y*-axis it represents liquid volume in milliliters (mL). Dots represent the number of licks occurring during the drinking bout.

**Figure 10:**
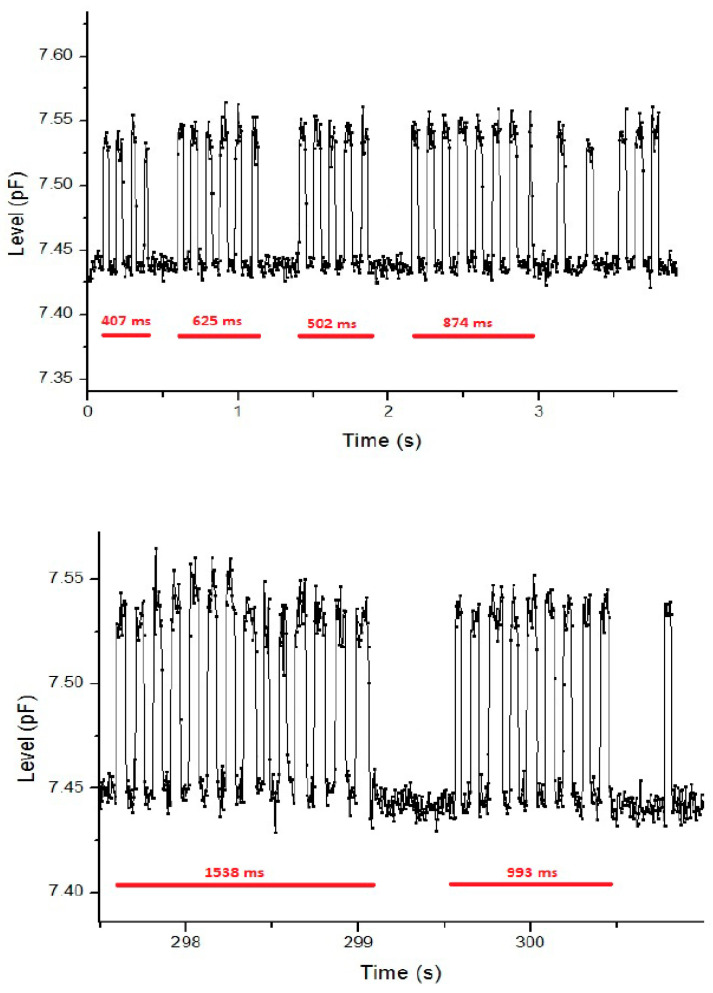
Acquisition of a microstructural analysis of lickings performed in CD1 mice. The duration of each burst is highlighted in red.

**Behavioral tests:** The programmable system on the chip device was tested in mice and rats housed in their standard home cages. The bottles used for experiments were of standard 250 mL capacity, square in shape, and with a stainless-steel spout. Mice and rats used for experiments were housed in twos. They were kept on a 12 h dark/light cycle from (9:00–21:00). The drinking behavior in mice was monitored for 5 consecutive days (Figure 9) and for 3 days in rats (Figure 10). The liquid-level sensor was used to gather data and it was connected to a PC through a USB cable with dedicated software designed for data collection. In mice, results demonstrate a gradual decline in liquid volume over time. The fluctuation in the graph (Figure 9) shows a period of higher and lower drinking activity, reflecting the dark active and light inactive periods, respectively. Average daily consumption recorded by the bottle was 6.8 mL/mouse and 26 mL/rat. This drinking level is consistent with data reported in the literature, indicating that daily water consumption for the mouse is around 3–7 mL [8]. In mice, recording of the licking pattern allowed evaluation of microstructural analysis of drinking (Figure 10). The duration of each burst is highlighted in red. The top graph shows the sharp peaks, which represents individual licking events over time, with the red bar indicating distinct licking bouts or clusters of licking activity. The bottom graphs show more detailed information of the licking activity during the specific time window (298–300 s), where longer and shorter licking bouts (1538 mms and 993 ms) are clearly visible.

**Figure 11:**
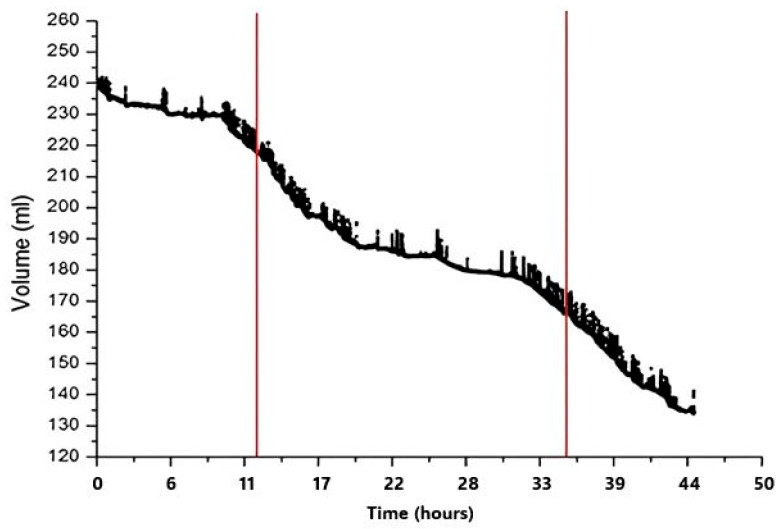
Drinking pattern in rats during the 3 days of testing. Midnight is shown by red vertical lines. The time is plotted on *x*-axis (in hours), whereas on *Y*-axis it represents liquid volume in milliliters. Dots represent the number of licks occurring during the drinking bout.

In rats, water consumption was recorded for 3 consecutive days (Figure 11). The results showed an average daily water consumption for rats of 26 mL, which corresponds to the daily drinking level classically reported for rats [9]. Also, in this case, the fluctuation in water consumption reflects periods of higher and lower activity occurring during the dark active and light inactive periods of the daily dark/light phases, respectively. When an animal licks the bottle spout, the capacity sensing system records an alteration from coupling with the animal’s body fluids. The electronic system can detect this little parasitic capacitance by producing a tiny spike in the readout data. The study of these spikes allows the analysis of the microstructure of lickings. As shown in Figure 10, the licking frequency was approximately 9.2 Hz, which means each lick occurs approximately every 108 ms, and the duration of a single licking event is 54 ms on average. The duration of each bout varies, as noted by the annotated times (407 ms, 625 ms and 874 ms) reflecting natural variability in the animals’ licking behavior. The consistent spacing of the peak confirms the rhythmic nature of licking behavior, while the stable single amplitude suggests uniform licking strength across events.

**Figure 12:**
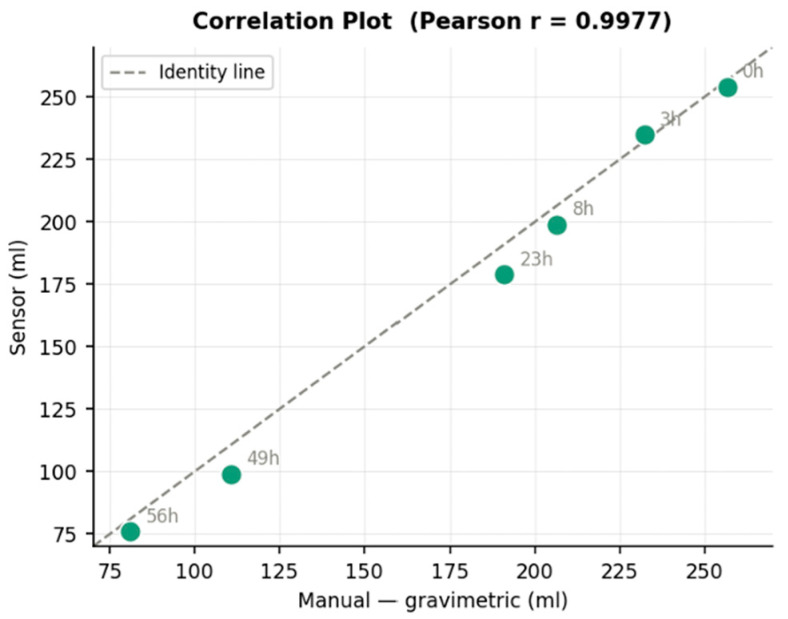
Correlation plot obtained, comparing automated and gravimetric analysis.

**Figure 13:**
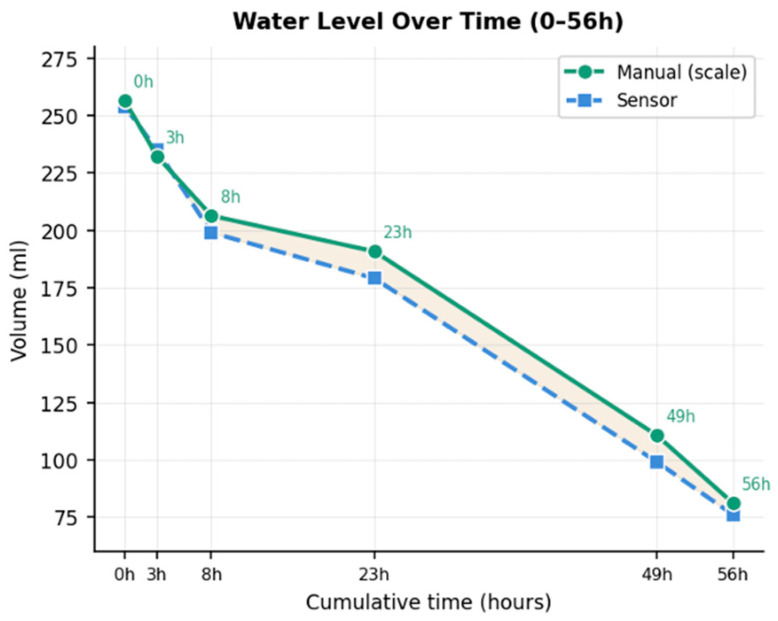
Comparisons between sensor reading and gravimetric measurements during 56 h of observation.

**Comparison with gravimetric measurements**: The gravimetric validation of the device was carried out for a period of 56 h. During this period, to confirm the quality of the automated measurements, gravimetric values were taken by removing the bottle from the device and weighing it with a standard laboratory scale. For this purpose, an additional two rats housed in a common cage were used. Pearson correlation analysis (Figure 12) showed a high linear correlation with the gravimetric reference methods (r = 0.9998), reflecting consistent volume tracking. Throughout the evaluation period (Figure 13), gravimetric and automated measurements followed the same pattern with the highest degree of overlap from 0 to 8 h.

## 5. Discussion

The major result of this work was the realization of a new electronic device, based on capacitance sensing to monitor drinking behavior in laboratory animals. From a technical standpoint, the effectiveness of this apparatus relies on the high sensitivity and stability of the capacitive sensing method used to detect changes in liquid level and tongue–spout interactions. Capacitive sensors measure variations in the dielectric field surrounding the electrodes, which shift predictably as the fluid volume inside the bottle changes or when the animal’s tongue contacts the insulated spout. The Out-of-Phase electrode configuration, together with shielding elements, minimizes electrical noise and external interference, thereby ensuring reliable detection of drinking. This design allows continuous and precise monitoring of liquid consumption, enabling the resolution necessary to capture rapid, millisecond-scale licking events. The combination of high sampling frequency and noise-resilient signal acquisition is what makes the device capable of extracting microstructural parameters of drinking, such as interlick intervals or burst duration, with a level of accuracy comparable to specialized lickometers, but within a more flexible, low-cost, and home cage-compatible system [12,13,14]. Moreover, if the animal is micro-chipped, the system can be expanded by integrating an RFID detector capable of identifying individual subjects as they interact with the bottle spout. This enhancement would allow precise attribution of drinking events to specific animals even when several are housed together in the same cage. The ability to track individual drinking patterns within a socially housed environment represents a major methodological advancement, as most existing drinkometer systems require single housing to avoid data confounds, thereby introducing stress and altering natural behavioral rhythms [16]. Social isolation is well known to affect a wide range of physiological and behavioral processes in rodents, including anxiety levels, circadian activity, reward sensitivity, and consummatory behavior, and can therefore constitute a confounding variable in studies aimed at addressing motivational or hedonic states. By contrast, an RFID-enabled system allows the preservation of the social context in which rodents normally live, while still providing high-resolution, individualized intake data. This capability opens new avenues for experimental designs that examine how social hierarchy, environmental enrichment, or competition influence fluid consumption, relapse-like drinking, or responses to pharmacological treatments. Thus, the integration of RFID technology not only increases the scalability of the device but also significantly enhances its ecological validity, enabling researchers to obtain more reliable and biologically meaningful measures of ingestive behavior under naturalistic group-housing conditions.

The possibility to analyze the microstructure of drinking is an additional element to consider as it allows the possibility to conduct experiments to determine how specific pharmacological or brain manipulations can affect drinking behavior. These studies are particularly important when, for example, experiments are designed to study reward responses following drinking of palatable sweet solution, alcohol or flavored fluids. Changes in the microstructure of drinking provide a powerful window into the motivational and reward processes that govern ingestive behavior [17]. Parameters such as latency to the first lick, burst duration, inter-bout interval, licking frequency, and total number of bouts are tightly regulated by neural circuits involved in incentive salience and hedonic valuation. An increase in bout size or licking frequency, for example, is typically associated with heightened motivation or increased palatability of the ingested solution, whereas fragmented bouts, prolonged pauses, or reduced lick rates often reflect decreased motivational drive or the emergence of negative affective states. These microstructural features therefore act as sensitive behavioral markers capable of revealing subtle shifts in reward processing induced by pharmacological manipulations, genetic modifications, or alterations in internal states (e.g., hunger, thirst, stress). By enabling high-resolution quantification of these parameters directly in the home cage, the present device allows researchers to detect motivational and hedonic changes that may not be evident from total intake alone, thereby improving the interpretability of studies investigating the reinforcing properties of palatable solutions, alcohol, or other rewarding or aversive fluids. The drinking device described here can also be used to aid the continuous monitoring of animals’ health status. In rodents, one of the earliest and most reliable indicators of malaise or disease onset is the alteration in drinking and feeding behavior [18].

Because these changes often precede overt clinical signs, an automated system capable of detecting reductions in intake, fragmentation of drinking bouts, or abnormal circadian patterns provides a valuable early-warning tool. Early identification of such deviations facilitates timely veterinary intervention, improving both animal welfare and experimental reliability by preventing the progression of undetected illness. Beyond general health surveillance, the system offers important applications in the monitoring of reproductive physiology. For instance, parturition and subsequent lactation in pregnant dams are characterized by a marked and abrupt increase in water consumption associated with milk production [10]. Automatically tracking these changes enables caretakers and researchers to pinpoint the timing of birth more accurately, verify lactation status, and identify dams that may be experiencing postpartum complications. These health- and reproduction-related uses are particularly relevant in large rodent breeding facilities, where staff must monitor hundreds of cages simultaneously and where continuous human observation is impractical. Integrating this device into routine husbandry procedures would therefore support both welfare-oriented surveillance and efficient colony management by providing real-time behavioral indicators directly within the home cage.

Several limitations of the present study should be acknowledged. First, in the absence of a direct comparison between the proposed prototype and commercially available systems, it is not possible to fully evaluate the relative advantages and disadvantages of our device, although experimental validation and calibration data support its reliability and accuracy.

Second, while the device demonstrated stable performance over short- to mid-term recording periods (3–5 days) and across species, including higher intake volumes in rats, longer-term validation would further strengthen confidence in its robustness. Extended testing was limited by battery duration, which constrained prolonged continuous monitoring. Ongoing technical improvements are aimed at increasing battery life to enable longer-term recordings. A further limitation is that comprehensive evaluation of the system would require testing with multiple fluids characterized by different viscosities and dielectric properties, as these factors may influence capacitive measurements. Future studies will be specifically designed to address these aspects and provide a more extensive validation of the device’s performance.

Finally, when we compared gravimetric and sensor-based measurements, despite the high degree of correlation (Pearson coefficient = 0.9977), at later timepoints we detected an underestimation of the volume measured with the sensor compared to the manually collected weight. This phenomenon was not observed during the first 8 h but became evident when the consumed volumes between measurements were larger at later timepoints.

This discrepancy is likely due to the capacitance-based operating principle of the sensor. Each time the bottle was removed from the holder for gravimetric measurements and then repositioned, fluid movement within the bottle altered the capacitance readings. In addition, minor but unavoidable fluid spillage may have further affected the measurements. We expect that this issue will not occur when bottles are not removed for gravimetric comparisons. Nonetheless, this observation highlights a critical characteristic of our sensing system that must be taken into account when it is used in experimental settings.

In summary, our study describes a novel, automated, and cost-effective system for monitoring home cage fluid consumption in rodents. The results demonstrate that, in addition to providing accurate measures of fluid intake, the system enables microstructural analysis of licking behavior, making it suitable for sophisticated investigations of ingestive behavior.

## 6. Patents

This study was conducted using our patented liquid monitoring system, International Patent No. PCT/EP2019/066453, to investigate ingestive behaviors in rodents and to support preclinical research on drinking patterns in laboratory animals.

## Data Availability

The original contributions presented in this study are included in the article material. Further inquiries can be directed to the corresponding authors.

## Abbreviations

The following abbreviations are used in this manuscript:

pF	Picofarad
SDA	Serial Data
SD	Storage Data
USB	Universal Serial Bus
